# Evaluation of school food policies for secondary schools in Europe: Results for health, acceptance, and affordability from a scoping review

**DOI:** 10.1111/obr.13911

**Published:** 2025-02-25

**Authors:** Nadia Blecha, Janina Meuer, Wiebke Hübner, Lara Christianson, Maike Wolters, Heide Busse, Antje Hebestreit, Sarah Forberger

**Affiliations:** ^1^ Department Prevention and Evaluation Leibniz Institute for Prevention Research and Epidemiology ‐ BIPS Bremen Germany; ^2^ Research Group: Interventional Health Care Research (IHCR) German Center for Neurodegenerative Diseases (DZNE) Greifswald Germany; ^3^ Department of Health Science University of York York UK

**Keywords:** obesity prevention, school food, school food standards, secondary schools

## Abstract

**Introduction:**

All European Union (EU) countries have established national school food policies. However, evaluations of those policies for secondary schools remain limited. This scoping review aims to synthesize the evidence of school food policies in secondary schools on child health, acceptance, and affordability in the EU, UK, Switzerland, Norway, and Iceland.

**Methods:**

The scoping review adheres to the PRISMA‐ScR guideline. Searches were conducted in four databases from 2000 to September 2023 without language and methods restrictions following a published protocol. After a two‐stage screening process, reviewers extracted data using a standardized and predefined coding scheme.

**Results:**

The search identified 185 records with *N* = 10 articles meeting the inclusion criteria (*n* = 7 UK, *n* = 1 each in Norway, Sweden, and Portugal). Among the included articles, *n* = 7 addressed school meal acceptance, *n* = 6 addressed health impacts, and *n* = 3 addressed affordability. Findings indicate low acceptance rates of school meals. Results of several studies showed that the reformulated menus did not meet nutritional standards and were not accepted because of taste, quality, and pupils' different food preferences. Affordability was reported as a barrier across the three articles addressing this topic.

**Conclusion:**

The existing literature highlights challenges in interpreting the impact of school food policies on health, acceptance, and affordability. Further research is needed to strengthen the methodological approaches and increase the evidence to inform policy development and implementation.

## 1 INTRODUCTION

In recent decades, overweight and obesity have emerged as significant public health concerns among children and adolescents in Europe.^1^ Shifting lifestyles influence dietary patterns, including changes in eating habits and increased consumption of ultra‐processed foods with higher‐energy density^2^ put children at risk for metabolic disorders^3^ and affect overall health.^4^ As around 30% of foods and drinks children consume daily are consumed in schools,^5^ this setting constitutes an essential part of the daily nutritional provision for some pupils.^6^ A Cochrane review as early as 2007 found that school meals positively influenced schoolchildren's physical and psychosocial health and positively affected school attendance and mathematics performance.^7^ The European Union (EU) published the Action Plan on Childhood Obesity 2014–2020, emphasizing the significance of the school environment and school meals.^8^ Schools have been recognized as a potential key setting in promoting a healthy diet and lifestyle,^9^ primarily through providing healthy food via canteens or cafeterias^10^ and thereby influencing children's academic performance. The EU action plan underlines the vital role of school food provision in fostering a healthier environment for children.^8^

European countries adopted different approaches to school food policies and their implementation. All EU member states, plus the United Kingdom (UK), Norway, and Switzerland, have established a national school food policy.^9^, ^11^ In 2014, these school food policies were either written as a separate policy (65%) or embedded in accompanying policies like education or health, with 18 policies setting mandatory standards and 16 voluntary guidelines (mandatory: Bulgaria, Croatia, Czech Republic, Estonia, Finland, France, Greece, Hungary, Latvia, Lithuania, Romania, Slovakia, Sweden, Slovenia, and UK; voluntary: Austria, Belgium, Cyprus, Denmark, France, Germany, Ireland, Italy, Luxembourg, Malta, Netherlands, Norway, Poland, Portugal, and Switzerland).^11^ Therefore, school food policies are heterogeneous and differ strongly in scope, aims, and implementation. Among countries evaluating school meals, 74% employ metrics for outcome evaluation, such as food provision, school food uptake, or child nutrition.^9^

However, evidence on health outcomes remains limited because of a lack of (long‐term) evaluations.^12^ Further, aspects that can hinder the successful implementation of a school food policy that later impact evaluation outcomes include acceptance and affordability of school meals. Children and adolescents mostly prefer foods with higher amounts of sugar and fat.^13^, ^14^ Previous studies have shown that changes in school meals toward meeting nutritional standards might not lead to a higher acceptance by older children and adolescents.^15^, ^16^ For example, in the UK, the introduction of minimum nutritional standards for school meals led to a significant improvement in the quality of food and an increased variety of products. Unfortunately, the new meals were less popular than traditionally prepared school lunches, largely because of the removal of high‐fat and high‐sugar foods and changes in preparation methods.^17^ Even if a school meal adheres closely to national recommendations, and thus accounts for culturally typical flavors and preparation methods, its health benefits may be diminished if pupils refuse consumption or cannot afford it as cost can be a significant factor influencing pupils' decisions for or against consuming school meals.^18^ These findings highlight the critical influence of both cost and taste in determining the success of school food policies.^17^, ^19^ While international studies suggest that school food policies can result in limited short‐term changes in specific dietary behaviors, including higher fruit consumption and lower intake of fats and sugar‐sweetened beverages,^20^, ^21^, ^22^ secondary schools are rarely the focus of study. A recently published study focusing on UK's secondary schools aimed to assess compliance with the school food standard legislation in English secondary schools and to explore the impact of the school food standard (SFS) legislation on pupils' nutritional intake.^23^ The authors examined three different types of SFS categories (lunchtime SFS, outside of lunch SFS, and additional whole day SFS) and found that lunch provision compliance with the national school food standards in secondary schools was the highest (median: 81.3%). However, on levels of compliance with their two identified types of standards, compliance was lower for standards restricting high‐fat, sugar, and energy‐dense items (median: 26.1%) than standards promoting dietary variety (median: 92.3%).^23^

To address this gap, this scoping review aims to synthesize the evidence and assess the scope of the literature on school food policies targeting school lunches in secondary schools in terms of impact on health (any reported health outcome), acceptance by different stakeholders, and affordability in the EU, UK, Switzerland, Norway, and Iceland. Further, the implementation strategies reported in those studies will be collected.

## 2 METHODS

### 2.1 Design

A scoping review approach was chosen as we explored a broad research question and mapped the existing literature on school food policy in secondary schools. It allows us to gain an overview of the field, identify knowledge gaps, and determine the scope of future research efforts. The scoping review adheres to the Preferred Reporting Items for Systematic Reviews and Meta‐Analyses (PRISMA) extension for Scoping Review (ScR) guidelines. The protocol was published beforehand.^24^ Eligibility for inclusion in the scoping review was determined based on the PICO (population, intervention, context/setting, outcome) criteria.

We included studies involving children and adolescents attending secondary schools (International Standard Classification of Education [ISCED] level 2 and level 3)^25^ aged between 10 and 18 years from European countries encompassing all European Union member states and the UK, Norway, Switzerland, and Iceland. As students finish the ISCED level, generally at 18 years, the age was set between 10 and 18 years. Studies including special needs schools and research papers addressing primary and secondary schools were included, where results were distinguishable between primary and secondary schools. For data analysis, only the data on secondary schools were extracted.

We included only studies that analyzed national school food policies. Following the definition of Lobczowska et al.,^26^ policies were defined “as actions developed and implemented to achieve specific goals within a society, with national or regional governments participating in the development and/or implementation of these actions.” Transferred to our scoping review, the definition is operationalized as follows: we included studies evaluating state‐ or sub‐state‐level nutrition policies and standards across all policy levels (local, regional, and national). The policies can be laws, guidelines, standards, or recommendations, but the institution issuing the policy must be a governmental body. Only studies targeting lunches served in cafeterias or canteens were considered. We excluded studies analyzing breakfast clubs; homemade lunch boxes; and policies on snacks, beverages, and vending machines. Further, studies reporting intervention projects were not included as the focus was on policies.

Outcomes extracted were health (any health outcome reported), acceptance, and affordability. Further, we extracted any data on the implementation strategy reported.

We included primary studies utilizing any design (qualitative and/or quantitative methods), while gray literature was omitted. We included any design, as qualitative or quantitative research can answer the research question.

### 2.2 Search methods

The search strategy was developed with an experienced information research specialist (LC) following an iterative technique adapted from JBI^27^, ^28^ (search query Appendix S1). First, the search terms for the concepts were developed and tested in MEDLINE. To cover the concepts of school meals, secondary school pupils, health outcomes, acceptance, and affordability, suitable keywords, synonyms, and MESH terms were combined to generate a structured search. The test results were analyzed, and the search strategy was further refined to enhance the precision of the search. The final search strategy was then adapted for the other databases (Appendix S1). Relevant studies were systematically sought in the following electronic databases: MEDLINE, PsycINFO, CINAHL, and Web of Science. The searches were conducted without language restrictions from 2000 until September 20, 2023. We included studies from 2000 onwards to account for the evolution of school nutritional policies over the past two decades.

Articles were imported into EndNote 20, where duplicates were identified and removed. The remaining articles were imported into Covidence, where additional duplicates were removed. Two independent reviewers screened both titles/abstracts and full texts. Articles not available in full text in English or German were excluded during the full‐text screening process and reported accordingly. Conflicts were discussed and solved by consensus.

### 2.3 Data extraction

Two independent reviewers systematically extracted data from the final articles. A data extraction sheet was developed in Microsoft Excel and pilot‐tested based on three included studies to ensure standardization of the data extraction procedure. The following characteristics of the included studies were extracted: first author, corresponding author, year of publication, country, study title, the aim of the study, study design, framework, policy, target group (name of the target, number of participants, control group, age, school age, grade, and gender), setting (description of setting, number of schools/canteens, and kind of schools), data collection (method, number of data collection timepoints, the time between data collection, follow up, methods of follow‐up, caterer involved, characteristics of the caterer, how often was the caterer approached, canteen/school kitchen involved, characteristics, how was caterer researched, and how often), health (outcomes and effects), acceptance (outcomes and effects), affordability (outcomes and effects), and implementation (what was assessed, how was it assessed, were effects reported, effect) (Tables 1 and 2).

**TABLE 1:** Description of characteristics of articles included.

Study/article characteristics		*N* = 10
Year of publication (range)		2006–2022
Country of study, *n*, multiple answers possible		
	United Kingdom	7
	Norway	1
	Portugal	1
	Sweden	1
Methodology, *n*		
	Quantitative	4
	Qualitative	5
	Mixed methods	1
Framework used, *n*		
	Yes	4
	No	6
What was addressed, *n*, multiple answers		
	Acceptance	7
	Health	6
	Affordability	3
Policy, *n*		
	National	5
	Constituent country policy	4
	County policy	1
Number of schools (range)		1–64
Target, *n*, multiple answers		
	Pupils	7
	Government administration	1
	Caterer staff	1
	Teachers	2
	Country	1
	Principals	1
	Meals	1

**TABLE 2:** Overview of all articles included.

Author, year, country	Study design Framework yes/no (if yes: name)	Research subject Number of research subject Age Number of schools	Intervention involved	Aim of the intervention Method of intervention Number of data collection Time between data collection Follow‐up	Policy Policy level	Caterer involved in evaluation	Health	Acceptance	Affordability	Implementation
Addis, 2019 United Kingdom	Qualitative, focus group Yes: Implementation Model	Pupils *N* = 52 nr^a^ 4 schools	No	NA NA NA NA NA	Appetite for Life action plan Constituent country (Wales)^b^	Yes	No	Yes (new menus do not align with food preferences, do not conform to peer norms)	No	No
Addis, 2019 United Kingdom	Qualitative, focus group No	Government administration, caterer staff, teachers *N* = 13 Nr^a^ 4 schools	No	NA NA NA NA NA	Appetite for Life action plan in Constituent country (Wales)^b^	No	Yes (more healthy food, more variation)	Yes (fear that students would struggle with new regulations, teachers welcoming to improved quality and range of food)	No	No
Ensaff, 2013 United Kingdom	Quantitative, cross‐sectional, surveys No	Pupils *N* = 2660 11–18 years 2 schools	No	NA NA NA NA NA	Nutritional Standards and Requirements for School Food, 2008 National	No	No	Yes (nutritionally sound dishes not popular, free school meal students choose healthier options, potential variations because of age)	No	No
Gould, 2006 United Kingdom	Quantitative, cross‐sectional, surveys and visual recording No	Pupils *N* = 74 11–12 years 3 schools	No	NA NA NA NA NA	Department for Education and Skills [DfES] Nutritional Guidelines (2001) National	No	Yes (no healthier food choices, new menus do not meet nutrition standards, girls eat healthier meals, meals in private schools are the healthiest)	No	Yes (high socio‐economic deprivation is negatively connected to food provision; cost for food higher at private school, possibly because of financial resources of caterer)	No
Gorelova, 2019 UK, Sweden	Qualitative, policy analysis No	Country policies NA NA NA	No	NA NA NA NA NA	National	No	Yes (high‐quality school meals)	Yes (reasons for not eating at the school canteen were an unsatisfactory quality of school dinners, poor menu choices, getting used to the type of dishes, school meals not being healthy enough or too boring, a lack of space for eating, an unpleasant situation in the canteen; noise, din, and a large number of peers; Friends do not eat in a school canteen either, this is a chance to have a break outside our school, we want to be outside, I am uncomfortable eating a homemade lunch packed by my parents in front of all other people.)	Yes (1/4 of students cannot afford school lunches)	Yes (only mentioned indirectly within *t* text)
Holthe, 2011 Norway	Qualitative, interviews Yes: Ecological Model	Pupils, teacher, principals *N* = 27 Nr^a^ 3 schools	No	NA NA NA NA NA	Norwegian national guidelines National	No	Yes (bought food is less healthy)	Yes (taste and look important; lack of variety and unpredictable availability are barriers)	No	Yes (barriers and facilitators)
Nelson, 2007 United Kingdom	Quantitative, cross‐sectional data analysis No	Pupils *N* = 713 4–18 years Nr^a^	No	NA NA NA NA NA	Department for Education and Skills [DfES] Nutritional Guidelines (2001) National	No	Yes (nutrients of school meals below recommendations, strong variation among nutrients and ways of school meal administration [free school meals] and gender)	No	No	No
Rito, 2020 Portugal	Quantitative (salt analysis), pre‐post analysis with follow‐up No	Meals *N* = 39 NA 10 kitchens/25 schools	Yes	Reduce salt in school meals Evaluating school meals, creating new school meals, post intervention samples Two 1 year No	“National Program for the Promotion of Healthy Eating” National	Yes	Yes (less salt in soup and per meal serving, salt reduction in almost all schools)	No	No	No
Ryan, 2022 United Kingdom	Qualitative, focus group Yes: Socio‐ecological model (SEM), food choice process model (FCPM)	Pupils *N* = 28 13–14 years 1 school	No	NA NA NA NA NA	UK school food policy (Department for Education), Childhood Obesity Action Plan (Department of Health and Social Care) National	No	No	Yes (buying food outside more convenient, pupils prefer little time‐consuming food and food provision procedures, good taste and look important)	Yes (healthier meal not chosen because of higher cost)	No
Townsend, 2011 United Kingdom	Mixed methods Yes Socio‐ecological model (SEM)	Pupils *N* = 6693 11–16 years 64 schools	No	NA NA NA NA NA	Welsh Network of Healthy School Schemes (WNHSS) Constituent country (Wales)	No	No	Yes (nr^a^)	No	No

^a^ nr = not reported.

^b^ UK consists of four constituent countries: England, Scotland, Wales, and Northern Ireland. Each of these constituent countries has its areas of policy responsibility and, therefore, its policies in certain areas, such as education, health care, and social policy. These countries can develop their own policies tailored to their specific needs and circumstances while still maintaining an overarching, unified policy for the entire UK.

### 2.4 Data analysis and presentation

Descriptive statistical analysis (e.g., country of study, methodology, framework used, and what was addressed) was undertaken to provide a concise overview of the study characteristics (Table 2). A narrative summary was drafted to illustrate how the results align with the review's objectives, incorporating qualitative and quantitative synthesis approaches. Data were summarized through thematic content analysis, wherein findings are categorized into thematic clusters based on the outcomes (health, acceptance, affordability, and implementation). We did not perform an overall assessment of the strength of the evidence, as the primary aim of the scoping review did not involve evaluating individual study quality.

## 3 RESULTS

**FIGURE 1:**
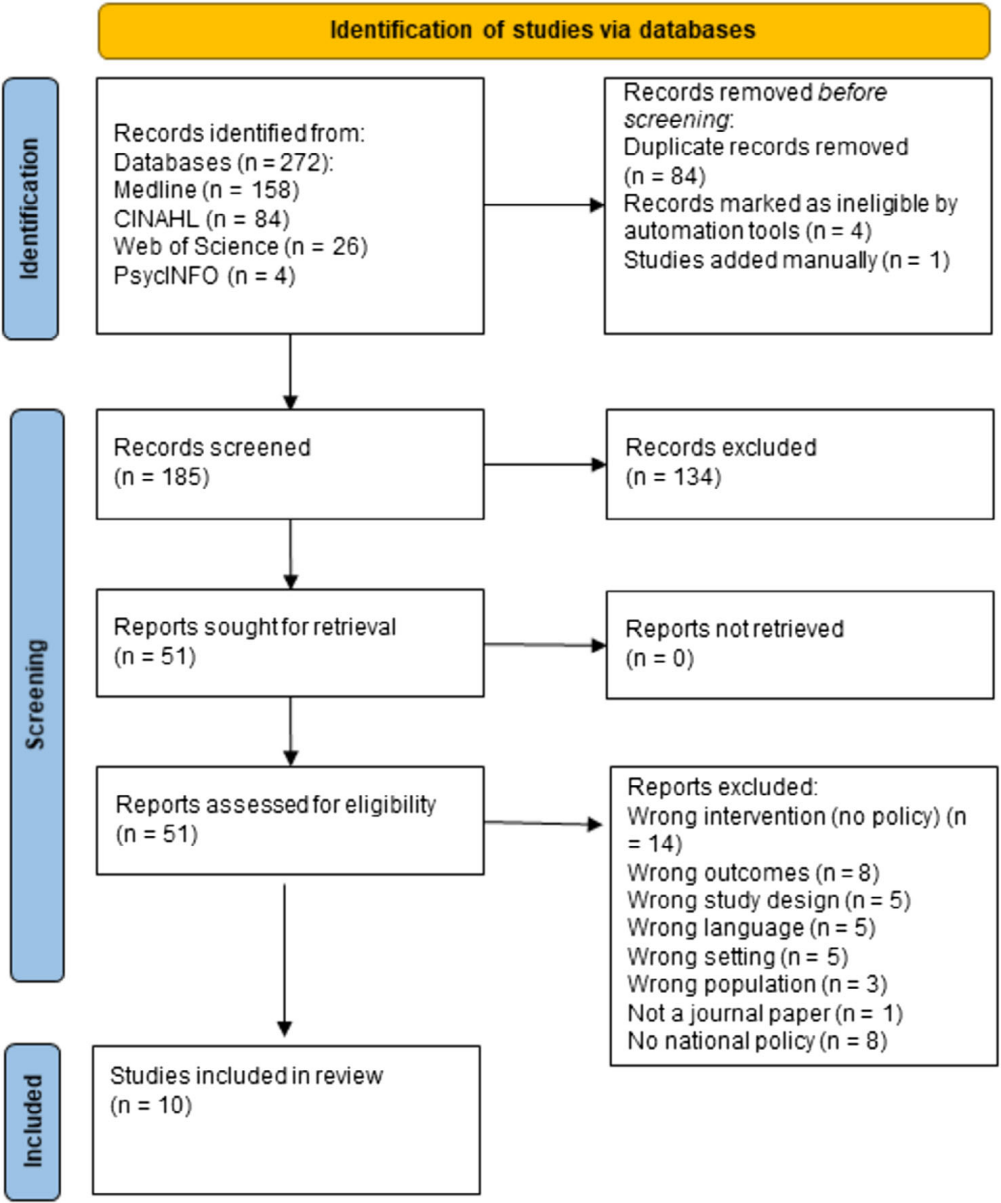
Prisma flow diagram.

Two hundred seventy‐two articles were identified in the four databases mentioned earlier. After removing duplicates, 188 articles were uploaded into Covidence software. After a second duplication check, the remaining 185 articles underwent title and abstract screening. Fifty‐one articles remained for full‐text review, resulting in data synthesis and extraction of 10 articles (Figure 1). Wrong intervention is referred to when focusing on an intervention or project but not on a national or subnational policy (*n* = 14). Eight studies had to be excluded as they did not report outcomes related to health, acceptability, or affordability. Additionally, eight papers were excluded because the papers reported no studies but traced historical developments, presented ideological concepts, or provided an overview of health promotion resources. Five studies were excluded because of language barriers (no full text in German or English). The wrong setting was defined as not a secondary school (*n* = 5) and the wrong population of school children not adhering to the age range of 10–18 years.

### 3.1 Characteristics

The characteristics of the included studies are described in detail in Tables 1 and 2.

Out of the 10 articles, five used a qualitative approach,^18^, ^29^, ^30^, ^31^, ^32^ four used a quantitative approach,^33^, ^34^, ^35^, ^36^ and one used a mixed methods approach, which combined semi‐structured interviews with questionnaires.^37^ Research in eight articles was conducted in the UK,^18^, ^29^, ^30^, ^31^, ^33^, ^34^, ^35^, ^37^ with other countries including Norway,^32^ Portugal,^36^ and Sweden.^31^ The articles referred to different policies at different levels. National policies were covered by seven articles (UK *n* = 5,^18^, ^31^, ^33^, ^34^, ^35^ Sweden *n* = 1,^31^ Norway *n* = 1,^32^ and Portugal *n* = 1^36^) where Gorelova et al. cover two countries (UK and Sweden).^31^ The other papers (*n* = 3) report on constituent country policies which are special to the UK.^29^, ^30^, ^37^ UK consists of four constituent countries: England, Scotland, Wales, and Northern Ireland. Each of these constituent countries has its areas of policy responsibility and, therefore, its policies in certain areas, such as education, health care, and social policy. These constituent countries can develop their own policies tailored to their specific needs and circumstances while still maintaining an overarching, unified policy for the entire UK.^38^ Among the 10 papers, five exclusively focused on one of the three outcomes (health^35^, ^36^ and acceptance,^18^, ^29^, ^33^, ^37^ whereas four addressed two or more^30^, ^31^, ^32^, ^34^ (Table 2).

Four studies used a framework to guide the study, namely the (socio‐)ecological model (*n* = 3),^18^, ^32^, ^37^ the food choice process model (*n* = 1),^18^ or the implementation model (*n* = 1).^30^

### 3.2 Health

Six articles covered the impact of national guidelines on health outcomes in secondary school children.^30^, ^31^, ^32^, ^34^, ^35^, ^36^ The sample size for pupils ranged from 13^30^ to 713,^34^ and the number of schools ranged from 3^32^, ^34^ to 25.^36^ Target groups were pupils analyzed either via interviews or school meals chosen,^28^, ^29^, ^31^, ^32^ in combination with interviews with school staff, catering staff, and government administration.^27^, ^29^ One paper reported analyzing the salt content of school meals.^36^ Countries covered were the UK,^30^, ^31^, ^34^, ^35^ Sweden,^31^ Norway,^32^ and Portugal.^36^

Addis et al.^30^ conducted a study examining the implementation of the Welsh school food policy “Appetite for Life” and its impact on the nutritional quality of school lunches in four Welsh schools. “Appetite for Life” is a set of nutritional guidelines in Welsh that aim to promote healthy eating and challenge traditional food choices, recognizing that nutritious meals are vital for students' health, learning, and behavior.^30^

Holthe et al. (2011) analyzed barriers to implementing the National Guidelines in Norway. They aim to ensure pupils' easy access to healthy meals, ensure adequate time and supervision for meals, and offer nutritious options like fruits, vegetables, and low‐fat milk while discouraging unhealthy items. The authors picked three case schools from 80 primary, 21 secondary, and 29 schools with predefined criteria (secondary schools, min. 250 pupils, inclusion of the project in the school's plan, the presence of a project group, and reported barriers in the baseline survey). Interviews were conducted with principals, project leaders, teachers, and pupils from tenth grade.^32^ To examine if the new school food had an impact on the healthiness of the food, both Addis et al. and Holthe et al. conducted qualitative interviews with staff members (administrative staff, principals, teachers, and caterers) from the UK schools^30^ and with staff members and pupils in Norway.^32^ School staff interviewed by Addis et al. reported an improved healthiness of school meals across all four schools. The interviewees found that introducing national guidelines resulted in healthier food options and increased variety in food choices. They reported an increase in overall quality and an increase in freshness of products.^30^ Holthe et al. suggested that, although pupils perceived school meals as healthier, offering higher‐grade students the option to purchase food outside the school undermines the concept of healthy school meals, as they observed increased consumption of unhealthy food when students did not eat at the school cafeteria.^32^

Gould et al.,^34^ Nelson et al.,^35^ and Rito et al.^36^ conducted quantitative research in the UK^34^, ^35^ and Portugal,^36^ either on pupils^34^, ^35^ and/or on school meal composition.^35^, ^36^ Gould et al. compared the meal choices of 74 children aged 11–12 years from three secondary schools in Sheffield, UK, with two public schools picked to reflect social and catering diversity and one all‐girls private school to represent variety in catering, although private schools do not have to adhere to statutory nutritional standards. The meals were compared based on the UK Department for Education and Skills (DfES) Nutritional Guidelines, which specifies offered food to meet nutritional guidelines, with two food items having to be served every day (starchy food, vegetables, fruit, milk and dairy products, and non‐dairy sources of protein) and recommendations for caterers on cooking methods. The daily lunch menus were analyzed for 5 days, ingredients and cooking methods were recorded, and a standard portion was weighed. Plates of all participants were photographed before and after meals, and food waste was estimated visually. The net amount of food was entered into a database to calculate nutrient intake. The lunch offered over 5 days was compared to the recommendations mentioned above from the DfES and scored with a scoring system. School menus offered by the private school were the healthiest and reached the highest scores, and the school with the highest socio‐economic deprivation scored the lowest. Gender differences in nutritional intake were observed, with boys consuming more total fat and girls having higher folate intake. School and gender interactions were observed for carbohydrates, starch, and calcium. Nutritional intake also differed between schools, with differences in monounsaturated fatty acids, polyunsaturated fatty acids, starch, calcium, and folate. They also found that school meals did not provide sufficient energy as required by the standards, and micronutrient intake was below the recommended amount for iron, folate, calcium, and zinc.^34^ Nelson et al.^35^ analyzed the nutritional components of school meals based on the same nutritional standard as Gould et al.^34^ They analyzed the 1997 NDNS data after the exclusion of unfit participants (based on health status and diet status) and then compared the remaining 713 participants from secondary schools with the 2004 surveys in the NDNS. Compared to data from 1997, pupils reported lower consumption of high‐fat main dishes; chips; other potatoes cooked in fat; and other cereals and higher consumption of vegetables, salads, sugar, preserves, and confectionery.^35^

Rito and colleagues analyzed the amount of salt in school meals by conducting pre–post analyses. Thirty‐nine school meal samples from 10 kitchens that served 25 schools were collected and analyzed for salt content. A typical school lunch in Portugal usually contains soup, bread, and a main dish. Together with public health professionals, nutritionists, a municipal food engineer, school cooks, teachers, and parents formed a working group to develop new school menus adhering to the national standards. Nutritionists also trained the schools' food handlers in topics such as food safety, cooking methods, and portion guides. After the adjusted school meals were introduced, samples were taken and analyzed. They found a significant salt reduction in 23 of the 25 schools involved. For the meals, the soup had a reduction of 34% of salt per serving, and an overall salt reduction of 23% for all three components was found.^36^

Gorelova et al.^31^ conducted a qualitative policy analysis comparing school food approaches in different countries (USA, UK, Sweden, and Russia). They reported that school lunches in Sweden usually contain two to three varieties of dishes, salads, a soup, and fruit or dessert with three main options (salads, main dishes, and vegetarian cuisine). They highlighted Sweden's provision of high‐quality school food and considered whether this contributed significantly to Sweden's lowest childhood obesity rates in Europe (18% compared to 23% in the EU). It was also pointed out that sugary drinks were prohibited, snacks (chips, cookies, and ice cream) were not for sale, and violations resulted in fines.^31^

In summary, the results were very heterogeneous; one study from the UK showed that school meals did not meet nutritional guidelines,^30^ whereas the Portuguese‐based study showed an improvement in school meal nutrition.^36^ One study referred to the existing concept with overall good results in health outcomes (Sweden).^31^ Several studies also reported challenges such as off‐campus food purchases,^32^ which jeopardize possible health advantages of the nutritionally adjusted lunches, and inconsistent nutritional content across different schools and countries.^34^, ^35^

### 3.3 Acceptance

Most of the articles focused on acceptance (*n* = 7), exploring perceptions among various stakeholders, including pupils,^18^, ^29^, ^31^, ^32^, ^33^, ^37^ government administrators,^30^ caterer staff,^30^ teachers,^30^, ^32^ principals,^30^, ^32^ and project leader responsible for the implementation of national guidelines.^32^ The sample size for pupils ranged from 13^30^ to 6693^37^ and number of schools ranged from 1^18^ to 64.^37^ In five articles, pupils were surveyed, by being asked to take part in focus groups,^18^, ^29^, ^32^ completing a questionnaire,^37^ or by having their purchases analyzed.^33^ Two studies surveyed school staff, including caterer staff and government administration.^30^, ^32^ Countries covered were the UK,^18^, ^29^, ^30^, ^31^, ^33^, ^37^ Sweden,^31^ and Norway.^32^

Addis et al. explored the nutritional changes in the school lunches in four Welsh schools that implemented the Welsh school food policy “Appetite for Life” and how secondary school pupils^29^ and school staff^30^ accepted it. Changes were introduced through new catering contracts. Pupils described the change in school meals as a reduction of high‐fat, high‐sugar food items.^29^ Chips were reduced to being served once a week, and recipes were adjusted to create healthier versions. This study defined acceptance as how well the adjusted school lunches aligned with pupils' food preferences.^29^ Ensaff et al. evaluated sales databases from two schools over 145 and 125 days, with 226.611 and 177.763 purchases, and compared pupils with and without access to the UK Free School meal program. The aim was to determine how pupils' food choices relate to current school food standards and how socio‐economic status influences this choice.^33^ Also, Addis et al.^29^ and Ensaff et al.^33^ conducted interviews and focus group discussions to understand students' perspectives on school food practices and choices in the UK. They found that new menus often did not align with students' food preferences and peer norms, leading to dissatisfaction among students. Ensaff et al. observed that dishes adhering to nutritional standards were less popular among students but more frequently chosen by free school meal pupils.^33^ Pupils in the focus groups conducted by Addis et al. reported low acceptance of the adjusted lunches. They perceived the new dishes as something they would instead be served at home and complained that they were not included in the decision‐making process of planning school meals, so they did not feel seen or heard and, therefore, refused to participate in the school lunches. In a second study.^29^ Addis et al.^30^ conducted semi‐structured interviews with government staff responsible for implementing food in school policy, teaching, and catering staff. Although the staff welcomed the changes in school lunch, they also acknowledged that the new menus were unpopular with the pupils. Catering staff complained about inflexible guidelines, which gave them no room to compromise on providing food that was more in line with pupils' preferences. One consequence was pupils' falling participation in school lunches. This was reinforced as two schools let pupils leave the school site for lunchtime starting from ninth grade on, which was allowed for practical reasons as the canteens of these schools could not cater to all of the pupils.^30^

Ryan et al. explored how adolescents made their food choices in a school environment because school food standards were introduced through the Childhood Obesity Action Plan and clashed with adolescents' usual food choices and preferences.^18^ Ryan et al.^18^ and Townsend et al.^37^ both used a socio‐ecological model (SEM) to examine dietary choices among pupils in the UK. While Ryan et al. conducted semi‐structured focus group interviews in one secondary school in Northern England,^18^ Townsend et al. utilized an already existing questionnaire, namely the 2005/2006 Health Behavior in School‐aged Children (HBSC) study. They included 64 schools in Wales and 6693 pupils for completeness of the questionnaires.^37^ Ryan et al. surveyed ninth graders on school food choices, and pupils' choices were influenced mainly by factors such as cost, taste, lunchtime duration, food availability, and social aspects. Pupils indicated that social aspects were the main driver for food choices, including wanting to spend more time socializing and less time queueing, queueing with friends rather than alone, and choosing lunch in line with their peers. The school environment gave the pupils more freedom to make unhealthy choices because the food environment at their homes was more strictly regulated by their parents. The interviews also revealed that, although pupils had some good knowledge of nutrition and health, most held misbeliefs, such as the belief that high‐sugar foods helped them meet their energy requirements and that healthy and unhealthy food balance each other out.^18^ Townsend et al. explain the variance in school food choices through an analysis informed by the socio‐economic level and explore which level had the most influence. They found that interpersonal factors (e.g., SES, what family/friends think they should eat for lunch, what friends eat for lunch) had a greater influence than intrapersonal characteristics (e.g., hours of TV viewing per week, on a diet, number of days eating breakfast/spend time after school with friends). School‐level organizational factors (e.g., school type, the school has a healthy eating policy, and the school has whole school healthy eating campaigns) were more able to explain unhealthy food choices. In contrast, community factors (e.g., number of students on school roll, SES percentage of students on free school meals, and links to the community regarding healthy eating) were able to explain variance in the dietary choice of food eaten throughout the day.^37^

Holthe et al. investigated the barriers and facilitators to implementing the Norwegian national guidelines for healthy school meals as perceived by principals, project leaders, teachers, and students.^32^ The analysis revealed several barriers to implementing healthy school meals, with distinct perspectives from students and staff. Four categories emerged: (1) Students noted that taste, lack of variety, and unpredictable availability of school food were key barriers, alongside concerns about hygiene and the appearance of food. Students also highlighted limited options compared to external food outlets, which they could access during lunch breaks. (2) Staff frequently mentioned insufficient resources, such as space and funding, as significant barriers. Running school canteens required teacher supervision and student labor, which diverted resources from educational activities. Students lacked the skills to manage the canteen effectively, limiting the quality and variety of healthy options. (3) Staff also expressed tension between prioritizing teaching responsibilities and implementing healthy school meal policies. Some felt promoting healthy eating should remain the responsibility of parents, while students faced challenges balancing canteen duties with academic commitments. (4) Students' ability to leave school premises during lunch encouraged purchasing unhealthy options. Extended lunch breaks without engaging in on‐site activities further exacerbated this trend, undermining compliance with healthy eating guidelines.^32^

These practical, organizational, and cultural barriers hindered the acceptance of healthy school meals. Students were primarily focused on food appeal and accessibility, while staff were more concerned with systemic resource constraints and goal conflicts.

For the UK, Gorelova et al. cited results from a telephone survey of 502 schoolchildren aged 11 to 16 years. Only 11% of the children interviewed indicated they had regular meals at the canteen. Of those children reporting to refuse to eat at the school canteen, common reasons for not eating at the school canteen in the UK included unsatisfactory food quality, poor menu choices, and an unpleasant cafeteria environment. Peer influence also played a significant role in students' meal choices; for example, pupils would instead opt out of eating at the cafeteria when their friends did not eat there.^31^ For Sweden, Gorelova et al. reported that while school meals are high‐quality, varied, and nutritious, they do not fully satisfy all students, mainly as they grow older. By the seventh grade, only 60% continue to eat in school, as older students prefer café breaks. The authors identified taste and presentation as crucial factors influencing acceptance for pupils. They also noted that the most important barriers are taste, lack of variety, and unpredictable availability. Other barriers mentioned were a perceived lack of hygiene and unpredictable opening hours. Some of the principals and project leaders and all teachers interviewed reported the implementation as too time‐consuming and argued that the responsibility of healthy food intake should belong to parents and not schools.^31^

Overall, while all studies focused on acceptance, they differed in their methods and the specific aspects of acceptance examined, highlighting the multifaceted nature of this topic and the diverse factors influencing students' perceptions of school meals. None of the above‐reported studies evaluated how the meals adjusted to meet the school food policies and nutritional guidelines were offered or how often they were offered or conducted a follow‐up.

### 3.4 Affordability

Three articles investigated the impact of the affordability of school meals on pupils.^18^, ^31^, ^34^ The sample size of pupils included ranged from 28^18^ to 74,^34^ and the number of schools ranged from 1^18^ to 3.^34^ The UK^20^, ^29^, ^32^ and Sweden^29^ are the countries covered.

Ryan et al. investigated the affordability of school meals. They found that high prices hindered accessing healthier food options.^18^ Similarly, Gorelova and colleagues reported that a quarter of students could not afford standard school meals in the UK.^31^ Further, Gould and colleagues^34^ discovered a negative association between high socio‐economic deprivation and nutrient intake in schools in the UK. They also noted that private schools had higher prices, likely because of higher ingredient prices (ingredient cost of 59 pence at the two state schools vs. £1 at the private school).^34^

### 3.5 Implementation strategies

In addition to the points mentioned, we were also interested in the reported implementation strategies, if provided, to understand how the policies were implemented by the various actors. Two articles were mentioned on implementation strategies. The studies covered different implementation aspects, analyzing implementation aspects of school food policies in the UK,^31^ Sweden,^31^ and Norway.^32^

Gorelova et al. focused on implementing national government programs in the UK and Sweden, mentioned the focus but specific information regarding implementation and implementation processes were not reported.^31^ Holthe et al. analyzed barriers to implementing national guidelines for school food policies in Norway. All staff groups perceived barriers related to implementation, particularly concerning resources and funding for running a canteen, for conflicting values and goals, as principals and teachers had to implement the national guidelines for school meals in addition to their regular work, and students had to leave their lessons to operate the canteen.^32^

## 4 DISCUSSION

This scoping review provides a synthesized overview of the evidence and the scope of the literature on school food policies targeting school lunches regarding health, acceptance, and affordability and reported implementation strategies in secondary schools in EU countries and the UK, Norway, Switzerland, and Iceland.

For health benefits, it is crucial that school food policies align with nutritional guidelines, a condition that was frequently not met in the studies reviewed.^32^, ^33^ Some studies have reported improvements in the health of the food provided. This mixed evidence highlights the need for further research, including comparisons between the guidelines and the food offered in schools. Such evaluations, potentially integrated into regular implementation assessments, could strengthen policy enforcement and improve the effectiveness of school food programs. Affordability emerged as a barrier in all three articles focusing on this outcome.^18^, ^31^, ^34^ Inadequate affordability hindered pupils' access to healthy food, particularly in cases where schools permitted older pupils to leave the school grounds during lunch breaks. Therefore, the government must ensure an adequate budget for providing healthy, tasty, and affordable school meals under consideration of all associated costs and contextual structures (such as time for eating or space). This kind of public health measure—in combination with, for example, education on diet and daily motivation for physical activity—can help to prevent subsequent costs of an unhealthy diet and obesity in youth and in later years.^39^ Also, state‐subsidized, mandatory school meals for all would also reduce inequality by decoupling access to school meals from parents' economic circumstances. However, this aspect is complex and requires further investigation. Studies have also reported that, particularly in older children, offering free school meals does not necessarily lead to higher food acceptance. Acceptance among older children might be low as they primarily value autonomy in food choices.^29^ While school meals at low or no cost offer positive outcomes, including improved food security, reduced financial stress for families, and opportunities for nutritional education, challenges remain. When designing free access to school meals, the issue of exclusion and discrimination must be considered.^17^ There is a need for more studies that address these aspects, particularly regarding the social dynamics and potential stigma associated with receiving free meals and how these factors may influence food acceptance and overall policy effectiveness.

All articles addressing acceptance reported low acceptance rates of school meals following the national guidelines among secondary school pupils because of unattractive and unpalatable food,^18^, ^29^, ^30^, ^31^, ^32^, ^33^, ^37^ including the food's color, texture, and taste. Additionally, the influence of peers on food choices and organizational barriers like long queues, limited eating time, and an unpleasant eating environment were commonly reported factors.^18^, ^31^, ^37^ Some research suggests that pupils felt excluded from the decision‐making process and, therefore, opted out of eating at the school canteen.^29^ Further, our results show that more attractive meal options and a pleasant atmosphere with sufficient eating time are required to increase the acceptance of healthy school meals.^29^ The studies did not report how often meals adapted to adhere to the new school food policies for meal composition were offered, how they were offered, or if a follow‐up was conducted. This would have been important information, as one study found that the mere exposure effect can increase children's acceptance of vegetables.^40^ The mere exposure effect is a psychological phenomenon where repeated exposure to an object refines the individual's relationship to it.^39^ In this study, children were more inclined to like the previously disliked vegetable when offered for 14 consecutive days compared to the control group.^40^ This would mean that for our study, novel or revised school meals following the national guidelines should be offered several times until a shift in the acceptance rate can be measured. More research is needed to explore how often new or adapted meals must be provided in the school setting to change acceptance in combination with the fitting evaluation framework.

Although our study indicates that school food policies mostly do not lead to appealing, enjoyable, and popular school meals, we assume that our findings may not fully reflect how food policies impact secondary school pupils in European countries. Existing literature rarely focuses on secondary schools. One possible reason for this could be the implicit assumption that students in secondary schools would respond in the same way as those in primary schools and that no adaptation of the implementation is needed for older students. However, this is merely a hypothetical assumption, and further research is required to explore this aspect more thoroughly. A recent study, published late in 2024, assessed compliance with the SFS legislation in English secondary schools and explored its impact on pupils' nutritional intake. The findings of this study align with those from our scoping review. Not all schools fully adhere to the national SFS, with the least compliance observed in restrictions on high‐fat, high‐sugar, and energy‐dense foods and drinks, suggesting that these standards are particularly challenging to implement in the secondary school context. Moreover, the level of compliance showed little association with pupils' nutritional intake at lunchtime.^23^ The studies reporting health outcomes interpreted health as a change in lunch options. None of the studies examined health outcomes in children, which can likely be attributed to the study designs and the absence of repeated surveys. Studies examining possible health benefits associated with successfully implemented school meal standards are lacking. They would improve our understanding of public health policy interventions that promote health in the school setting. More research, especially using pre–post study designs (measured shortly before a policy change and after the new policy was enacted and implemented) and designs with repeated measurements considering school meal acceptance combining various methods, are needed to understand better and gain a deeper insight into why pupils may not choose healthier school meal options and how different choices impact their health. Studies with repeated offerings of nutritionally reformulated meals could measure acceptance in children and adolescents over time. Another approach could be using synthetic control methods to evaluate the effect of school food policies. Also, the combination with nutritional analyses of the meals served in a mixed‐methods design approach could prove advantageous in this regard, as such analyses are less susceptible to biases such as recall bias or availability bias, which may affect questionnaires on food consumption. Standardizing the measurement of health outcomes would enable policymakers and stakeholders to effectively compare various programs and tailor their policies to align with more successful initiatives, if necessary. Standardizing health measurements would enhance the understanding of results and allow for better conceptualization, enabling effects to be more accurately attributed to specific policies. In our scoping review, none of the studies reported on body mass index (BMI), health markers (such as metabolic markers), or weight. A similar approach can be taken concerning *acceptance*. Studies would benefit significantly if the concept of acceptance were clearly defined and operationalized. In the context of school food policies, acceptance is a complex, multi‐dimensional construct that plays a crucial role in determining the success of interventions like school meal programs. By operationalizing this concept, researchers can ensure consistency in measurement and produce more robust, comparable results across different studies. Acceptance can be approached from several angles. Behavioral acceptance might be assessed through participation rates, such as the number of students choosing the meals or consuming them fully. Attitudinal acceptance could be measured by evaluating students' or parents' perceptions of the food, including whether they like it, view it as nutritious, or believe it promotes a healthy lifestyle. Social acceptance involves examining how the broader school community, including teachers and staff, perceives the meals and whether the policy is seen as beneficial or culturally appropriate. Lastly, cultural acceptance addresses whether the meals align with the dietary preferences or needs of diverse student populations, an essential factor in ensuring that the policy is inclusive and relevant to all students.

The scarcity of the literature, coupled with the variety in methods, settings, and target groups, complicated the interpretation and underscored a significant gap in the existing academic literature.

### 4.1 Limitations

Studies that did not explicitly name the research objective as governmental actions or policy and only mentioned the approaches as interventions were excluded during the screening process. For instance, some papers may have referred to policies as “welfare interventions” or omitted terms such as “policy” or “school food policy” entirely. Additionally, studies where the link to governmental approaches was not clearly articulated in the title or abstract may have been misclassified or inadvertently excluded. This underscores a notable gray area in policy analysis and highlights the lack of standardized terminology for describing such interventions within the literature. Another limitation of this scoping review is the lack of in‐depth analysis of the policies. While we have provided a brief description of the policies, including their names and the classification of each as national or subnational (Table 2) as reported in the texts, it was not feasible to conduct a comprehensive policy analysis given the resource constraints within our team. As such, we did not examine key elements such as the scope and aims of the policies, the issuing and leading institutions for implementation, inter‐ministerial cooperation, or whether an implementation plan is attached to the policy. This limitation reflects a broader issue in policy studies, where such detailed information is often inadequately reported or unavailable. Additionally, there is a lack of a unified framework or guidelines regarding the essential aspects that should be reported in policy analyses. This gap in standardized reporting further complicates the process of obtaining and assessing these critical policy details.

We initially planned to search the websites of the EU and WHO Europe and those of the ministries of European countries for potential further studies. Deviating from the protocol because of limited resources and language barriers, this could not be carried out as initially planned, and therefore, the overview is based solely on the scientific literature.

### 4.2 Implications

Scientific research should monitor and evaluate the implementation of school meals based on national guidelines. Standardized and evidence‐informed procedures, guided by implementation evaluation frameworks, would be especially beneficial to facilitate comparisons between different programs across Europe and within individual countries' schools and allow for evidence‐informed and context‐specific policies based on the success of similar programs in comparable structures.^36^ This approach would facilitate the comparison of different policies and their outcomes and inform the decision‐making process.

It is crucial to recognize that this demographic comprises adolescents who, while capable of taking responsibility for their health, still require various suitable options to guide their decision‐making toward healthier alternatives. Moreover, concerted efforts are essential to ensure that healthy food options in schools are nutritious, affordable, and inherently appealing to pupils, fostering their natural preference for these choices. Factors such as providing sufficient time to eat and creating a pleasant dining environment, including considerations like canteen design, furniture, background noise, and lighting, play a crucial role in enhancing the overall dining experience. Access to healthy school meals should be equitable for all pupils, ideally facilitated through state programs. It is widely recognized that providing healthy school meals is crucial to ensure pupils receive the necessary nutrients for optimal academic performance and overall healthy development and to help bridge the inequality gap in access to nutritious food and to counteract the “hidden hunger.”

## AUTHOR CONTRIBUTIONS

The corresponding author attests that all listed authors meet authorship criteria and that no others meeting the criteria have been omitted. Antje Hebestreit, Maike Wolters, Heide Busse, and Sarah Forberger conceived the idea and contributed to the manuscript's writing and revision. Janina Meuer, Nadia Blecha, Sarah Forberger, and Wiebke Hübner developed the research questions and study methods. Nadia Blecha and Lara Christianson developed the search strategy. Janina Meuer, Nadia Blecha, and Wiebke Hübner screened and extracted the data. Nadia Blecha drafted and edited the manuscript with Antje Hebestreit, Maike Wolters, Heide Busse, and Sarah Forberger, providing critical revisions. All authors read and approved the final version of the manuscript.

## CONFLICT OF INTEREST STATEMENT

The authors have nothing to report.

## Supporting information


**Data S1.** Supplementary Information.
