# Supplementary material for: Evaluation of school food policies for secondary schools in Europe: Results for health, acceptance, and affordability from a scoping review
Source: Obes Rev. 2025 Feb 25;26(7):e13911. doi: 10.1111/obr.13911 (PMC12137045; doi:10.1111/obr.13911)
Supplement: Supplementary file 1 — Data S1. Supplementary Information. [file OBR-26-e13911-s001.pdf]

Title: Evaluation of School Food Policies for Secondary Schools in Europe: Results for Health, Acceptance, and Affordability from a Scoping Review

Author
Dr. Sarah Forberger
forberger@leibniz-bips.de; ORCID: 0000-0002-7169-675X
Leibniz Institute for Prevention Research and Epidemiology - BIPS GmbH
Department of Epidemiological Methods and Etiological Research
Achterstrasse 30, 28359 Bremen, Germany
Tel.: +49 (0)421 218-56907
Fax: +49 (0)421 218-56941

Appendix 1: Search Strategy

Medline via PubMed (Date: 20. September 2023)

**Search line**	**Search terms**	**Results**
**1**	("School meal*"[Title/Abstract] OR "school lunch*"[Title/Abstract] OR "school cafeteria*"[Title/Abstract] OR "school canteen*"[Title/Abstract] OR "School menu*"[Title/Abstract] OR "School food*"[Title/Abstract] OR "School feeding*"[Title/Abstract] OR "food suppl*"[Title/Abstract])	15,899
**2**	(lunch[MeSH Terms]) OR (food services[MeSH Terms])	16,103
**3**	#1 OR #2	30,364
**4**	("food-based standard*"[Title/Abstract] OR "food-based guideline*"[Title/Abstract] OR "school food standard*"[Title/Abstract] OR "school food guideline*"[Title/Abstract] OR "food-based recommendation*"[Title/Abstract] OR "food guideline*"[Title/Abstract] OR "food polic*"[Title/Abstract] OR "nutrition standard*"[Title/Abstract])	1,703
**5**	(nutrition policy[MeSH Terms])	12,773
**6**	#4 OR #5	13,839
**7**	(Accept*[Title/Abstract] OR compliance[Title/Abstract] OR cooperat*[Title/Abstract] OR attendance[Title/Abstract] OR participat*[Title/Abstract] OR attitude [Title/Abstract])	1,588,665
**8**	(Health*[Title/Abstract] OR "health outcome*"[Title/Abstract] OR nutrition*[Title/Abstract] OR "healthy foo*"[Title/Abstract] OR "healthy choic*"[Title/Abstract] OR "healthy consumption"[Title/Abstract] OR "eating health*"[Title/Abstract] OR "nutrition benefit*"[Title/Abstract] OR "diet qualit*"[Title/Abstract] OR "nutrition improvement*"[Title/Abstract] OR "food educat*"[Title/Abstract] OR "diet inequalit*"[Title/Abstract])	3,949,983
**9**	affordab*[Title/Abstract] OR cost*[Title/Abstract] OR "economic viability" [Title/Abstract] OR sustainabilit*[Title/Abstract]	866,935
**10**	(teen*[Title/Abstract] OR adolescen*[Title/Abstract] OR youth[Title/Abstract] OR boy[Title/Abstract] OR boys[Title/Abstract] OR girl*[Title/Abstract] OR pupil* [Title/Abstract] OR student* [Title/Abstract])	1,011,261
**11**	adolescent[MeSH Terms]	2,221,400
**12**	#10 OR #11	2,794,887
**13**	("Secondary school*"[Title/Abstract] OR "secondary education*"[Title/Abstract] OR "middle school*"[Title/Abstract] OR "high school*"[Title/Abstract] OR "upper school*"[Title/Abstract] OR "junior high school*"[Title/Abstract])	63,789
**14**	Schools[MeSH Terms]	146,826

**15**	#13 OR #14	198440
**16**	(Austria[Title/Abstract] OR Belgium[Title/Abstract] OR Bulgaria[Title/Abstract] OR Croatia[Title/Abstract] OR Cyprus[Title/Abstract] OR "Czech Republic"[Title/Abstract] OR Denmark[Title/Abstract] OR Estonia[Title/Abstract] OR Finland[Title/Abstract] OR France[Title/Abstract] OR Germany[Title/Abstract] OR Greece[Title/Abstract] OR Hungary[Title/Abstract] OR Iceland[Title/Abstract] OR Ireland[Title/Abstract] OR Italy[Title/Abstract] OR Latvia[Title/Abstract] OR Lithuania[Title/Abstract] OR Luxembourg[Title/Abstract] OR Malta[Title/Abstract] OR Netherlands[Title/Abstract] OR Norway[Title/Abstract] OR Poland[Title/Abstract] OR Portugal[Title/Abstract] OR Romania[Title/Abstract] OR Slovakia[Title/Abstract] OR Slovenia[Title/Abstract] OR Spain[Title/Abstract] OR Sweden[Title/Abstract] OR Switzerland[Title/Abstract] OR "United Kingdom"[Title/Abstract] OR England[Title/Abstract] OR Scotland[Title/Abstract] OR Wales[Title/Abstract] OR "Northern Ireland"[Title/Abstract] OR Britain[Title/Abstract] OR Scandinavia*[Title/Abstract] OR Europe*[Title/Abstract])	1,101,020
**17**	(Austria[MeSH] OR Belgium[MeSH] OR Bulgaria[MeSH] OR Croatia[MeSH] OR Cyprus[MeSH] OR "Czech Republic"[MeSH] OR Denmark[MeSH] OR Estonia[MeSH] OR Finland[MeSH] OR France[MeSH] OR Germany[MeSH] OR Greece[MeSH] OR Hungary[MeSH] OR Iceland[MeSH] OR Ireland[MeSH] OR Italy[MeSH] OR Latvia[MeSH] OR Lithuania[MeSH] OR Luxembourg[MeSH] OR Malta[MeSH] OR Netherlands[MeSH] OR Norway[MeSH] OR Poland[MeSH] OR Portugal[MeSH] OR Romania[MeSH] OR Slovakia[MeSH] OR Slovenia[MeSH] OR Spain[MeSH] OR Sweden[MeSH] OR Switzerland[MeSH] OR "United Kingdom" [MeSH] OR England[MeSH] OR Scotland[MeSH] OR Wales[MeSH] OR "Northern Ireland" [MeSH] OR "Scandinavian and Nordic Countries" [MeSH] OR Europe[MeSH])	1,552,456
**18**	#16 OR #17	2,092,213
**19**	#12 OR #15	2,883,139
**20**	#3 AND #6 AND (#7 OR #8 OR #9) AND #18 AND #19	161
**21**	#3 AND #6 AND (#7 OR #8 OR #9) AND #18 AND #19 Filters: from 2000 - 2023	158

PsychInfo (Date: 20. September 2023)

Sea rch line	Search terms	Resu lts
**1**	("School meal*" or "school lunch*" or "school cafeteria*" or "school canteen*" or "School menu*" or "School food*" or "School feeding*" or "food suppl*").ti,ab.	1,97 3
**2**	("food-based standard*" or "food-based guideline*" or "school food standard*" or "school food guideline*" or "food-based recommendation*" or "food guideline*" or "food polic*" or "nutrition standard*").ti,ab.	255
**3**	(Accept* or compliance or cooperat* or attendance or participat* or attitude).ti,ab.	602, 853
**4**	(Health* or "health outcome*" or nutrition* or "healthy foo*" or "healthy choic*" or "healthy consumption" or "eating health*" or "nutrition benefit*" or "diet qualit*" or "nutrition improvement*" or "food educat*" or "diet inequality").ti,ab.	884, 882
**5**	(affordab* or cost* or "economic viability" or sustainabilit*).ti,ab.	140, 993
**6**	(teen* or adolescen* or youth or boy or boys or girl* or pupil* or student*).ti,ab.	957, 566
**7**	("Secondary school*" or "secondary education*" or "middle school*" or "high school*" or "upper school*" or "junior high school*").ti,ab.	1232 80
**8**	exp schools/	7949 0
**9**	7 or 8	182, 969
**10**	(Austria or Belgium or Bulgaria or Croatia or Cyprus or "Czech Republic" or Denmark or Estonia or Finland or France or Germany or Greece or Hungary or Iceland or Ireland or Italy or Latvia or Lithuania or Luxembourg or Malta or Netherlands or Norway or Poland or Portugal or Romania or Slovakia or Slovenia or Spain or Sweden or Switzerland or "United Kingdom" or England or Scotland or Wales or "Northern Ireland" or Britain or Scandinavia* or Europe*).ti,ab.	236, 665
**11**	6 or 9	1004 500
**12**	1 and 2 and (3 or 4 or 5) and 10 and 11	4
**13**	1 and 2 and (3 or 4 or 5) and 10 and 11 Filters: from 2000 - 2023	4

CINAHL (Date: 20. September 2023)

Sea rch Lin e	Search terms	Resu lts
**1**	TI (("School meal*" or "school lunch*" or "school cafeteria*" or "school canteen*" or "School menu*" or "School food*" or "School feeding*" or "food suppl*")) OR AB (("School meal*" or "school lunch*" or "school cafeteria*" or "school canteen*" or "School menu*" or "School food*" or "School feeding*" or "food suppl*"))	4293
**2**	(MH "Lunch+") OR (MH "food services+")	1196 0
**3**	S1 OR S2	1512 2
**4**	TI (("food-based standard*" or "food-based guideline*" or "school food standard*" or "school food guideline*" or "food-based recommendation*" or "food guideline*" or "food polic*" or "nutrition standard*")) OR AB (("foodbased standard*" or "food-based guideline*" or "school food standard*" or "school food guideline*" or "foodbased recommendation*" or "food guideline*" or "food polic*" or "nutrition standard*"))	800
**5**	(MH "Nutrition Policy+")	5700
**6**	S4 OR S5	6202
**7**	TI ((Accept* or compliance or cooperat* or attendance or participat* or attitude)) OR AB ((Accept* or compliance or cooperat* or attendance or participat* or attitude))	5330 49
**8**	TI ((Health* OR "health outcome*" OR nutrition* OR "healthy foo*" OR "healthy choic*" OR "healthy consumption" OR "eating health*" OR "nutrition benefit*" OR "diet qualit*" OR "nutrition improvement*" OR "food educat*" OR "diet inequalit*")) OR AB ((Health* OR "health outcome*" OR nutrition* OR "healthy foo*" OR "healthy choic*" OR "healthy consumption" OR "eating health*" OR "nutrition benefit*" OR "diet qualit*" OR "nutrition improvement*" OR "food educat*" OR "diet inequalit*"))	1588 056
**9**	TI (affordab* OR cost* OR "economic viability" OR sustainabilit*) OR AB (affordab* OR cost* OR "economic viability" OR sustainabilit*)	2365 48
**10**	TI ((teen* OR adolescen* OR youth OR boy OR boys OR girl* OR pupil* OR student*)) OR AB ((teen* OR adolescen* OR youth OR boy OR boys OR girl* OR pupil* OR student*))	4689 24
**11**	(MH "Adolescence+")	6020 57
**12**	S10 OR S11	8727 43
**13**	TI (("Secondary school*" OR "secondary education*" OR "middle school*" OR "high school*" OR "upper school*" OR "junior high school*")) OR AB (("Secondary school*" OR "secondary education*" OR "middle school*" OR "high school*" OR "upper school*" OR "junior high school*"))	3474 8
**14**	(MH "Schools+")	8377 5
**15**	S13 OR S14	1112 10
**16**	TI ((Austria or Belgium or Bulgaria or Croatia or Cyprus or "Czech Republic" or Denmark or Estonia or Finland or France or Germany or Greece or Hungary or Iceland or Ireland or Italy or Latvia or Lithuania or Luxembourg or Malta or Netherlands or Norway or Poland or Portugal or Romania or Slovakia or Slovenia or Spain or Sweden or Switzerland or "United Kingdom" or England or Scotland or Wales or "Northern Ireland" or Britain or Scandinavia* or Europe*)) OR AB ((Austria or Belgium or Bulgaria or Croatia or Cyprus or "Czech Republic" or Denmark or Estonia or Finland or France or Germany or Greece or Hungary or Iceland or Ireland or Italy or Latvia or Lithuania or Luxembourg or Malta or Netherlands or Norway or Poland or Portugal or Romania or Slovakia or Slovenia or Spain or Sweden or	3379 66

	Switzerland or "United Kingdom" or England or Scotland or Wales or "Northern Ireland" or Britain or Scandinavia* or Europe*))	
**17**	(MH "Austria+") OR (MH "Belgium+") OR (MH "Bulgaria+") OR (MH "Mediterranean Islands+") OR (MH "Czech Republic+") OR (MH "Denmark+") OR (MH "Estonia+") OR (MH "Finland+") OR (MH "France+") OR (MH "Germany+") OR (MH "Greece+") OR (MH "Hungary+") OR (MH "Iceland+") OR (MH "Ireland+") OR (MH "Italy+") OR (MH "Latvia+") OR (MH "Lithuania+") OR (MH "Luxembourg+") OR (MH "Netherlands+") OR (MH "Norway+") OR (MH "Poland+") OR (MH "Portugal+") OR (MH "Romania+") OR (MH "Slovakia+") OR (MH "Slovenia+") OR (MH "Spain+") OR (MH "Sweden+") OR (MH "Switzerland+") OR (MH "United Kingdom+") OR (MH "Great Britain+") OR (MH "England+") OR (MH "Scotland+") OR (MH "Wales+") OR (MH "Northern Ireland+") OR (MH "Europe+")	653149
**18**	S16 OR S17	792221
**19**	S12 OR S15	921978
**20**	S3 AND S6 AND (S7 OR S8 OR S9) AND S18 AND S19	84
**21**	S3 AND S6 AND (S7 OR S8 OR S9) AND S18 AND S19 Filters: from 2000 - 2023	84

World of Science (Date: 20. September 2023)

Search Line	Search terms	Results
**1**	(TI=(("School meal*" OR "school lunch*" OR "school cafeteria*" OR "school canteen*" OR "School menu*" OR "School food*" OR "School feeding*" OR "food suppl*"))) OR AB=(("School meal*" OR "school lunch*" OR "school cafeteria*" OR "school canteen*" OR "School menu*" OR "School food*" OR "School feeding*" OR "food suppl*"))	30229
**2**	(TI=(("food-based standard*" OR "food-based guideline*" OR "school food standard*" OR "school food guideline*" OR "food-based recommendation*" OR "food guideline*" OR "food polic*" OR "nutrition standard*"))) OR AB=(("food-based standard*" OR "food-based guideline*" OR "school food standard*" OR "school food guideline*" OR "food-based recommendation*" OR "food guideline*" OR "food polic*" OR "nutrition standard*"))	3059
**3**	(TI=((Accept* OR compliance OR cooperat* OR attendance OR participat* OR attitude))) OR AB=((Accept* OR compliance OR cooperat* OR attendance OR participat* OR attitude))	3078681
**4**	(TI=((Health* OR "health outcome*" OR nutrition* OR "healthy foo*" OR "healthy choic*" OR "healthy consumption" OR "eating health*" OR "nutrition benefit*" OR "diet qualit*" OR "nutrition improvement*" OR "food educat*" OR "diet inequalit*"))) OR AB=((Health* OR "health outcome*" OR nutrition* OR "healthy foo*" OR "healthy choic*" OR "healthy consumption" OR "eating health*" OR "nutrition benefit*" OR "diet qualit*" OR "nutrition improvement*" OR "food educat*" OR "diet inequalit*"))	4393671
**5**	(TI=((affordab* OR cost* OR "economic viability" OR sustainabilit*))) OR AB=((affordab* OR cost* OR "economic viability" OR sustainabilit*))	2547052
**6**	(TI=((teen* OR adolescen* OR youth OR boy OR boys OR girl* OR pupil* OR student*))) OR AB=((teen* OR adolescen* OR youth OR boy OR boys OR girl* OR pupil* OR student*))	1928835
**7**	(TI=(("Secondary school*" OR "secondary education*" OR "middle school*" OR "high school*" OR "upper school*" OR "junior high school*"))) AND AB=(("Secondary school*" OR "secondary education*" OR "middle school*" OR "high school*" OR "upper school*" OR "junior high school*"))	35764
**8**	#6 OR #7	1934929
**9**	(TI=((Austria OR Belgium OR Bulgaria OR Croatia OR Cyprus OR "Czech Republic" OR Denmark OR Estonia OR Finland OR France OR Germany OR Greece OR Hungary OR Iceland OR Ireland OR Italy OR Latvia OR Lithuania OR Luxembourg OR Malta OR Netherlands OR Norway OR Poland OR Portugal OR Romania OR Slovakia OR Slovenia OR Spain OR Sweden OR Switzerland OR "United Kingdom" OR England OR Scotland OR Wales OR "Northern Ireland" OR Britain OR Scandinavia* OR Europe*))) OR AB=((Austria OR Belgium OR Bulgaria OR Croatia OR Cyprus OR "Czech Republic" OR Denmark OR Estonia OR Finland OR France OR Germany OR Greece OR Hungary OR Iceland OR Ireland OR Italy OR Latvia OR Lithuania OR Luxembourg OR Malta OR Netherlands OR Norway OR Poland OR Portugal OR Romania OR Slovakia OR Slovenia OR Spain OR Sweden OR Switzerland OR "United Kingdom" OR England OR Scotland OR Wales OR "Northern Ireland" OR Britain OR Scandinavia* OR Europe*))	3273867
**10**	#1 AND #2 AND (#3 OR #4 OR #5) AND #8 AND #9	26
**11**	#1 AND #2 AND (#3 OR #4 OR #5) AND #8 AND #9 Filters: from 2000 - 2023	26
